# A Wide-Area Coverage 35 Gb/s Visible Light Communications Link for Indoor Wireless Applications

**DOI:** 10.1038/s41598-019-41397-6

**Published:** 2019-03-20

**Authors:** Hyunchae Chun, Ariel Gomez, Crisanto Quintana, Weida Zhang, Grahame Faulkner, Dominic O’Brien

**Affiliations:** 1 Department of Engineering Science, University of Oxford, Oxford, OX1 3PJ United Kingdom; 2 Microsoft Research Ltd, 21 Station Road, Cambridge, CB1 2FB United Kingdom; 3 Airbus UK, Filton Pegasus House, Aerospace Avenue, Filton, Bristol, BS34 7PA United Kingdom; 4 Deptment of Information and Telecommunication Engineering, Incheon National University, Incheon, 22012 Korea

## Abstract

Visible Light Communications (VLC) can provide both illumination and communications and offers a means to alleviate the predicted spectrum crunch for radio-frequency wireless communications. In this paper, we report a laser diode based white-light communications link that operates over a wide area and supports high data rates. The proposed system is a four-colour multiplexed high-speed VLC system that uses a microelectromechanical system (MEMS) mirror-based beam-steering. The system operates at record data-rates of more than 35 Gb/s (Bit Error Rate(BER) < 3.8 × 10^−3^) with a coverage area of 39 m^2^ at a link distance of 4 m. To the best of our knowledge this is the fastest VLC demonstration reported thus far. The paper also addresses issues of eye-safety, showing data rates of  more than 10 Gb/s are feasible.

## Introduction

Next generation communication technology is expected to bring orders of magnitude enhancement in data-rate compared with existing wireless communications^1,2^. New applications such as virtual reality (VR) and augmented reality (AR) will be enabled by high bandwidth connectivity, and the Internet of Things will create a demand for a network that can serve billions of devices. Outdoor field trials have been reported^2,3^, but considering that over 80% of mobile data traffic is indoors^4^, it is also crucial to build high capacity indoor network architectures providing multi Gb/s communications.

There is growing interest in indoor visible light communications (VLC) due to its potential to combine illumination and communications, and it provides ~THz of unlicensed bandwidth (BW), with a high degree of spatial reuse, and high security^5^. In 2012, a commercial white light emitting diode (WLED) based 1 Gb/s data transmission was demonstrated^6^. Three years later, 2 Gb/s was achieved using the WLED^7^. Although these commercial WLEDs illuminate the room, the data-rate is limited to a few Gb/s mainly due to the low BW of the source. Therefore, to obtain higher data-rate, multiple channel multiplexing techniques such as wavelength division multiplexing (WDM) were investigated. A WDM system achieving a 2.3 Gb/s using blue GaN µLEDs with two fast colour converters for green and red colours was demonstrated in 2015^8^. Later, references^9–11^ reported 5.6 Gb/s, 8 Gb/s, and 10 Gb/s, respectively, using WDM with three or four colour LEDs. Reference^12,13^ presented WDM with the use of RGB laser diodes (LD) demonstrating the rate of 8 Gb/s and 14 Gb/s, respectively. However, these high-speed systems provide only fixed point to point communication links. In order to make the high-capacity communication link shared flexibly with multiple users or equipment, it is essential to create a system that communicates with users across a wide area. Beam-steering of infra-red (IR) beam with a relatively small beam footprint has been investigated^14–18^. By steering the beam, this method can flexibly deliver a high capacity communication channel to end-users in a room-scale area. In 2001, reference^14^ suggested the use of a wavelength-dispersive optical element with fast tunable lasers, steering the angle by tuning the wavelengths of the incoming beam. Recently, reference^15^ demonstrated a Gb/s system using micro-electro-mechanical-systems (MEMS) mirror based beam-steering. References^16^ and^17^ showed 224 Gb/s and 416 Gb/s using a spatial light modulator (SLM) with 512 by 512 pixels. More recently in 2018, 112 Gb/s was achieved using a commercial camera lens and 2- dimensional fibre bundle from an arrayed waveguide grating router^18^.

Most of the reported high speed visible-light systems though have shown fixed point-to-point links with limited coverage. They used a simple direct modulation and detection and relatively cheap silicon receiver, achieving the maximum reported data-rate of ~10 Gb/s. The biologically friendly visible wavelength allows manufacturers, maintenance technician and end-users to have better controllability of the communication channel. Visible light systems can also provide lighting function.

Most of the IR beam-steering systems reported higher data-rate (~100 Gb/s) using relatively complex methods such as high bandwidth external modulation, polarisation control and high sensitivity InGaAs receivers often with optical amplifiers. The achieved high rates are also due to more relaxed eye safety limits in IR compared to visible wavelengths. Relatively high power is allowed because the retina is less vulnerable with IR light.

The use of steerable mirror and visible wavelength beams can be an alternative to the IR systems. It supports 5 G (eMBB: enhanced Mobile Broad Band)’s target peak data-rate of 10 s of Gb/s, and the requirement for VR. (6 degree of freedom (DoF) immersive experience through VR needs 5 Gb/s^19^.) The coverage and mobility are provided by beam-steering. In addition, the beam-steered visible wavelength system can still take advantage of the special features mentioned above, as opposed to its invisible counterpart.

**Figure 1:**
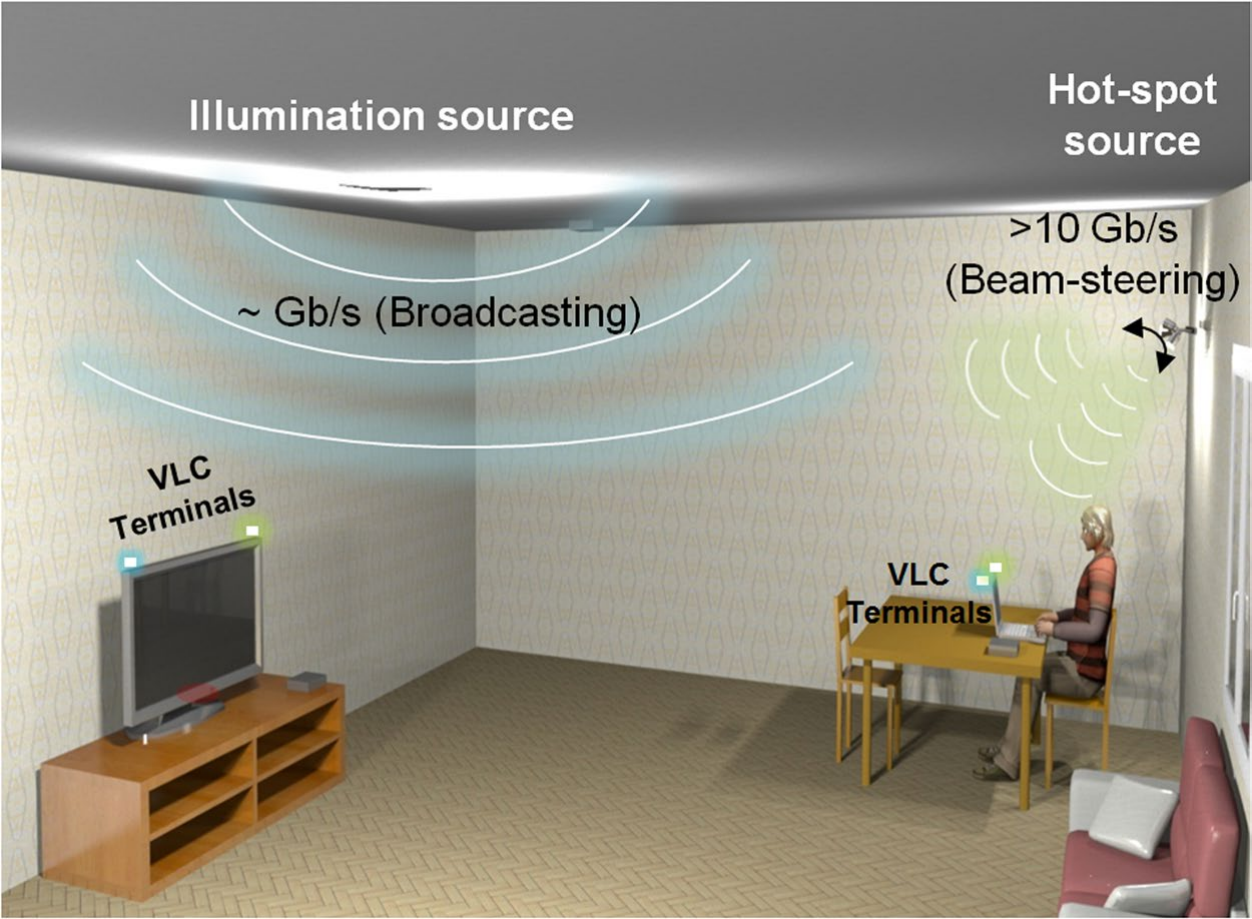
An indoor scenario for visible light communications with a few Gb/s broadcasting and >10 Gb/s hot-spot link.

Figure 1 shows a system that provides wide area coverage to users using room illumination (~Gb/s), and high capacity (>10 Gb/s) and wide coverage using a beam-steered link. In this paper we focus on the high capacity hot-spot design and report a four-colour white-light beam-steered optical link capable of providing such an ultra-high capacity link.

## Results

### System description

**Figure 2:**
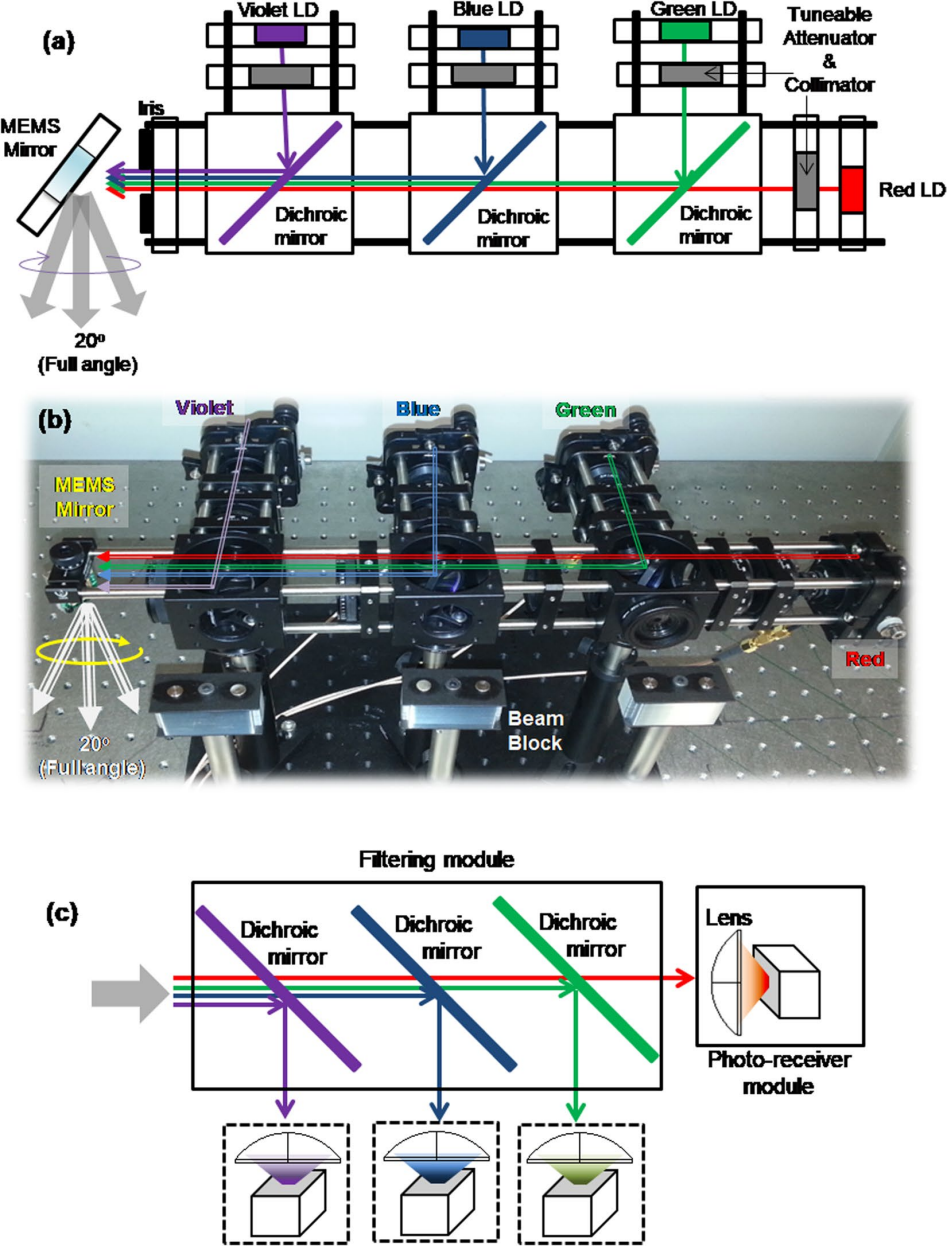
Four colour WDM VLC system incorporated with a MEMS mirror based beam-steering module. (**a**) Schematic and (**b**) set-up implementation of the transmitter and (**c**) the receiver structure.

Figure 2 shows a four wavelength multiplexed LD based VLC source, combined with a MEMS mirror based beam-steering module. First, beams from the red, green, blue, and violet LDs are collimated and combined into a single beam using three dichroic mirrors as shown in Fig. 2(a). The divergence of each beam can be independently adjusted depending on the desired beam characteristics, and each beam can be independently tuned to ensure the beams are co-aligned. Each beam path also incorporates an attenuator, based on two rotating polarisers, that allows the relative intensity of each colour component to be adjusted.

The co-aligned beams then pass through an iris, which ensures the beams are the same diameter, then into a beam-steering system. A MEMS mirror with an aperture diameter of 5 mm and a mechanical and optical steering range of +/−5 degree and +/−10 degree, respectively, is used to steer the beam, and a well-collimated beam with a minimum diameter of 10 mm at 4 m (divergence half angle <0.072 deg) is obtained from this system. Detailed information about the sources, optics, and the MEMS mirror is given in the methods section.

### Communication performance

An orthogonal frequency division multiplexing (OFDM) scheme is used to check the communication capacity of the proposed system^11^. OFDM is one of the most widely used modulation schemes, especially when pursuing high data-rate links since it allows adaptive bit and power loading to the sub-carriers according to the communication channel condition.

For OFDM, first, *M*-ary quadrature amplitude modulation (*M*-QAM) symbols are encoded from a distinct bit stream for each colour channel. An inverse fast Fourier transform (IFFT) is used to convert the *M*-QAM symbols allocated on different subcarriers to time-domain samples. The samples outside of the linear dynamic range set by each LD are clipped. Then, an arbitrary waveform generator (AWG) generates an analogue signal from the conditioned time samples, followed by a wide band high-power electrical amplifier. With the addition of DC bias, the analogue signal is applied to each LD. The LDs emit red, green, blue, and violet light with a wavelength distribution centred at 660 nm, 520 nm, 450 nm, and 400 nm, respectively. The driving condition can cause instantaneous fluctuation of an illumination level, but when DC-balanced modulation scheme (such as DCO-OFDM) is used, with a stable average current, the impact is negligible^20^.

The system shown in Fig. 2 then steers the resulting beam, which propagates to a receiver. To support the WDM configuration, optical band-pass filtering is performed using the filtering module similar to the colour combining unit in the transmitter, as shown in Fig. 2(c). A 2-inch diameter high numerical aperture lens is used to capture the filtered light onto a 1.4 GHz BW free-space photo-receiver. This is a valid approach as a negligible crosstalk is expected between the four implemented colour channels. Each channel is from a narrow linewidth LD with no spectral overlap, hence allowing for independent WDM characterisation as widely reported in literature^8–12^. (The specifications of LDs and the pass-bands configured by the dichroic mirrors are in the methods section.) Each separated and optical-to-electrical (O-E) converted signal is sampled by an 2.5 GHz BW oscilloscope for communication performance tests. Finally, MATLAB^®^ is used to process the captured electrical signal for decoding and analysing the channel adaptive optical OFDM.

The time taken to send one OFDM frame is determined by the total number of samples in one frame and the sampling rate. The total number of samples includes the IFFT (*N*_fft_) samples generated from the *M*-QAM symbols on the sub-carriers and the cyclic prefix samples (*N*_*c*_) dealing with the interference between frames and sub-carriers. The sampling rate is determined by over sampling rate (*f*_os_) and system sampling clock (*f*_clk_). Then, the frame time (*T*_f_) is calculated as

1 $${T}_{f}=({N}_{{\rm{fft}}}+{N}_{{\rm{c}}})\frac{{f}_{os}}{{f}_{clk}}[\sec \,.]$$

The system clock is normally fixed for a specific system. The oversampling rate is an integer value with which the modulation BW is adjusted. To maximize the use of all available resources within the modulation BW, a channel adaptive bit and power loading algorithm based on^21^ is used. For a given target bit error rate (BER) performance, the algorithm determines the number of bits on each sub-carrier with optimized power level, leading to the maximum total bits (*b*_*sum*_) maintaining the same total power $({P}_{RF}^{total})$.

2 $${b}_{sum}=\,\max (\sum _{k=1}^{\frac{{N}_{{fft}}}{2}-1}{lo}{{g}}_{2}{M}_{k})[{\rm{bits}}]\,{\rm{and}}\,{P}_{RF}^{total}=\sum _{k=1}^{\frac{{N}_{{\rm{fft}}}}{2}-1}{P}_{k}[{\rm{W}}]$$

where *M*_k_ is the optimized level of *M*-QAM symbols on *k*^th^ sub-carrier, which is set to 1 when the sub-carrier is determined to be empty by the algorithm. Then, the maximum data-rate (*DR*_max_) from a WDM link with a total optical power constraint $({P}_{optical}^{total})$ is given as

3 $$D{R}_{{\rm{\max }}}=\,\max (\sum _{\lambda =1}^{{N}_{wdm}}\frac{{b}_{sum,\lambda }}{{T}_{f,\lambda }})[b/s],\,{\rm{subject}}\,{\rm{to}}\,{P}_{optical}^{total}=\sum _{\lambda =1}^{{N}_{wdm}}{P}_{\lambda }[{{\rm{W}}}_{{\rm{optical}}}]$$

where *N*_*wdm*_ is the number of WDM channels, and the subscript *λ* is the index of each WDM channel.

**Figure 3:**
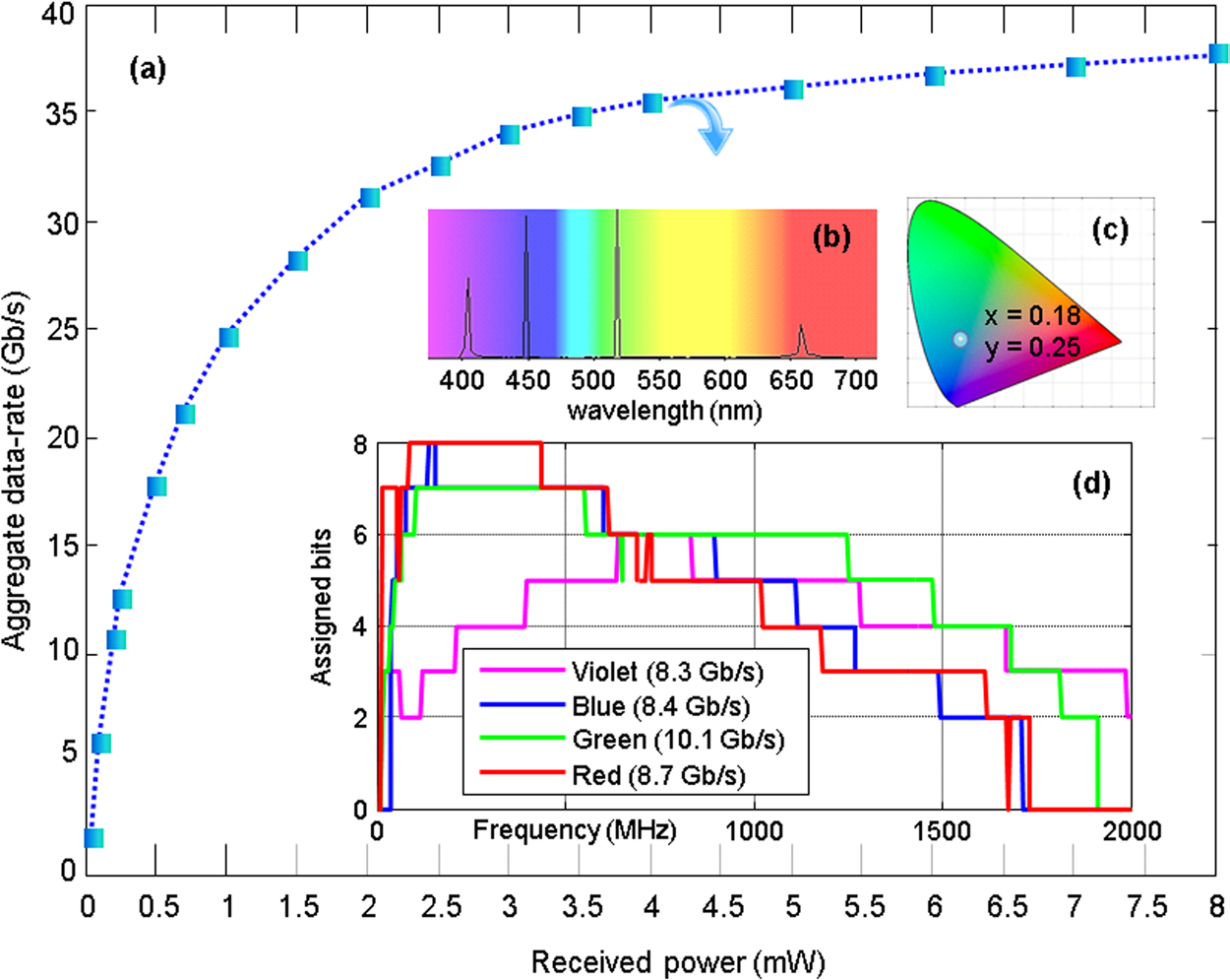
WDM results for maximum data-rate. (**a**) Received optical power in total vs. measured aggregate data-rate using channel adaptive OFDM, where the power proportion is adjusted to maximise the data-rate for a given power level. (**b**) Optical spectrum, (**c**) CIE colour coordinate, and (**d**) Assigned bits for each colour leading to 35.6 Gb/s (BER < 3.8 × 10^−3^).

Figure 3(a) shows the maximum data-rate achieved below the target BER of 3.8 × 10^−3^ from the four colour WDM system presented in Fig. 2. The target BER guarantees a reliable communication link by applying forward-error-correction coding with 7% overhead^22^. In the demonstration, due to the receiver aperture wider than input beam size from the line of sight pencil beam, the received power is equated with the transmitted power. The maximum achievable data rate depends both on the total power transmitted and the proportion of each colour used. The four colours are optimally mixed to achieve the highest data-rate given the same total power. The maximum data-rates logarithmically increase with increased the total received optical power, achieving 10.9 Gb/s at 0.2 mW, 21.2 Gb/s at 0.7 mW, and 35.6 Gb/s at 4 mW. The figure suggests that the rates could be further increased with greater numbers of WDM channels, as channel rates are saturated beyond 20 Gb/s. For instance, from 4 mW (35.6 Gb/s) to 8 mW (37.8 Gb/s), there is only a 2.2 Gb/s improvement. If the additional 4 mW is used for accommodating more WDM channels, the rate could become double depending on the source BWs and colour dependent property of the receiver. Figure 3(b–d) show the optical spectrum, CIE colour coordinate, and assigned bits for each colour, at the data-rate point of 35.6 Gb/s, respectively.

### Coverage

**Figure 4:**
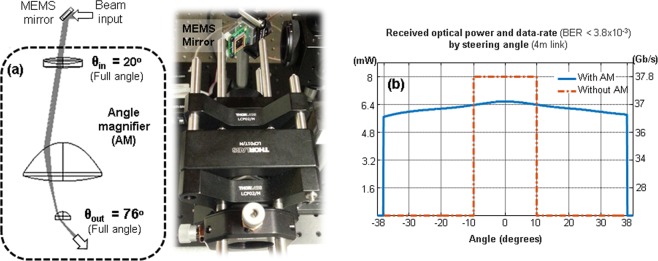
Coverage. (**a**) Angle magnifier module (**b**) received optical power and data-rate by steering the beam with and without the angle magnifier.

A MEMS steering mirror provides the coverage. The mirror’s optical beam-steering range of +/−10 degree can cover 0.35 × 0.35 m^2^ at 1 m and 1.4 × 1.4 m^2^ at a 4 m link distance. However, these areas can be insufficient for a full coverage of some indoor environments. Figure 4(a) shows an angle magnifier module located at the transmitter output. The module magnifies the pointing angle of the combined light. The angle magnification factor is defined by focal lengths of the lenses and the distances between them. Here, the parameters are optimized to achieve the magnification factor of 3.8, allowing a wide coverage of 6.25 × 6.25 m^2^ at 4 m link distance, but enlarging the spot size by a similar factor. Figure 4(b) shows the received optical power and the data-rate for different steering angles with and without the angle magnifier module. Without the angle magnifier, 8 mW (37.8 Gb/s) is received within +−10 degrees of beam-steering. With the use of angle magnifier, +−38 degrees with data-rates greater than 36 Gb/s are achieved, despite the relative loss of 1 to 1.5 dB due to the enlarged beam size while magnifying the angle. In this report, WDM capable hot-spot lighting system and its coverage are mainly proposed and examined. For a receiver alignment, a beam-steering unit similar to the one in the transmitter can be used. Or, fluorescent concentrators which selectively capture the incoming light by its absorption property in a non-imaging way^23^ can be applied.

### Considerations for lighting

LDs are considered efficient lighting sources with high luminous efficacy^24,25^. Also, LD based lighting systems can bring new lighting architectures with high controllability from low divergence^26^. For the use of LDs for lighting, however, eye-safety should be taken into account. Laser radiation hazards in the visible spectrum (400–780 nm) are dominated by retinal hazards, as described in the standard^27^. These are divided into photochemical and thermal hazards, which determine the accessible emission limits (AEL) for a given laser source. The AEL is the maximum allowed power for a laser to be part of a class, Class 1 being a safe source. The AEL not only depends on the wavelength of operation but also on the apparent source size and beam divergence. These two factors, together with the eye lens accommodation at the back of the retina determine the retinal image size. When the retinal image size becomes smaller, the hazard increases since the power density of the LD’s radiation on the retina becomes bigger. In this paper, the ‘small source’ condition has been applied to estimate the AEL for the emitted power, which is the most conservative safety calculation and worst-case scenario according to the standard^27^. In addition, the photochemical hazard limits the power level of blue wavelengths, and this often provides the most restrictive eye-safety condition for a safe white light generation.

**Figure 5:**
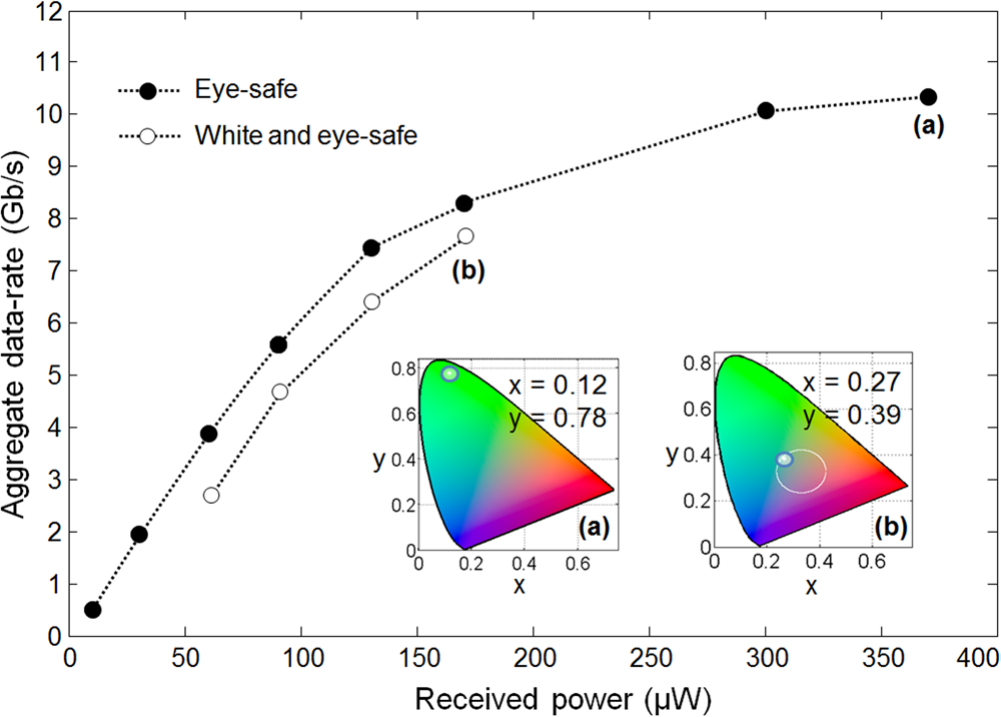
Examples. Maximum data-rate vs. received power for an eye-safe link, and white-light link with colour points in CIE coordinates at their highest data-rate of (**a**) 10.4 Gb/s and (**b**) 7.8 Gb/s, at BER < 3.8 × 10^−3^.

Figure 5 shows the maximum data-rates by received power levels for an eye-safe link meeting the criteria for Class 1^27^, with which all conditions of normal use are considered safe. The highest data-rate from the eye-safe case is 10.4 Gb/s at 370 µW, and the generated colour point at the rate is shown in Fig. 5(a). Achieving both higher data-rates and eye-safe operation is an area of current investigation, examining the geometrical modification of the sources (which controls the retinal image size) and the optimal wavelength distribution of the sources.

Figure 5a shows that the maximum eye-safe data rate is achieved using green light for the particular devices in this demonstration. However, white light can be preferred for general lighting. In this case, the white light requirements fix the proportion of RGBV colours. Then, the photochemical blue hazards become the most restrictive factor. Figure 5(b) shows the optimum point resulting in the highest data-rate satisfying both eye-safety and white-light constraint. Here, 20% deviation from the absolute white point (x = 0.33, y = 0.33) is used to approximate white-light boundary shown in^28^. The maximum data-rate for the eye-safe white-light cases is 7.8 Gb/s at 170 µW, and the generated colour point at the rate is shown in Fig. 5(b).

For general LD based illumination, there are still active and challenging topics of research. Illumination quality parameters such as colour rendering index (CRI) have to be further explored^20^. Discrete laser line sources are known to have a poor CRI, thus filling the uncovered spectrum with more WDM sources or with colour converted light from phosphor plates can help to improve this metric. Engineered extended sources defined by LD sources incident on a diffuser or a phosphor plate can release the limitation set by eye-safety regulation. Also, in depth study on laser products designed to function as conventional lamps is needed as its power emission limits are dictated by the lamp standard^29^.

## Discussion

In this report, a wide coverage visible light communication system with the maximum data-rate of 35 Gb/s (after FEC overhead reduction) was proposed and demonstrated. This can be potentially used for the 5^th^ generation communication system (or beyond) in indoor scenarios where a peak data-rate of >10 Gb/s is required. The achieved data-rates logarithmically increase with the received optical power. This suggests that the rates can be further increased with greater numbers of WDM channels when the rates are saturated, typically beyond 20 Gb/s. Beam-steering such links offers a means to provide wide area coverage, and a MEMS system with a demonstrated coverage area of 39 m^2^ at a link distance of 4 m has been shown. The data-rate and coverage presented in this report are the fastest and the widest coverage results in visible light communications to the best of authors’ knowledge and show the potential of such narrow beam links in providing future wireless communications. This 35 Gb/s demonstration aids to understand the limits of VLC communications channels using off-the-shelf laser diode components. Future work will focus on further engineering these sources to be more eye-safe, as well as improving the illumination quality for such laser-based systems. Also, comparative research on various beam-steering options for a practical smart hot-spot lighting configuration is of interest.

## Methods

The LDs used for violet, blue, green, and red colour channels are (Thorlabs) L405P20 with housings LTN330-A, PL450B with S1LM38, PL520 with S1LM38, and L658P050 with LTN330-A, respectively. L405P20 has a peak wavelength at 405 nm, linewidth less than 2 nm and beam divergence of 8.5° (parallel) and 12° (perpendicular). PL450B has a peak wavelength at 450 nm, linewidth of 2 nm and beam divergence of 11° (parallel) and 25° (perpendicular). PL520 has a peak wavelength at 520 nm, linewidth of 2 nm and beam divergence of 7° (parallel) and 22° (perpendicular). L658P050 has a peak wavelength at 658 nm, linewidth less than 2 nm and beam divergence of 10° (parallel) and 20° (perpendicular).

The collimator system includes 2-lens systems (352671-A and AL1210-A, Thorlabs) with XYZ positioning stage. This system was optimized to obtain +/−38 deg beam steering whilst maintaining the collinearity and minimizing the beam divergence. The dichroic mirrors used to combine the WDM channels are (Thorlabs) DMLP425, DMLP490, and DMLP550 with mounts B5C1, respectively. The combined beam is controlled by an iris (CP20S, Thorlabs) and is reflected by a MEMS mirror (Mirrorcle) with 5 mm diameter, +−10**°** steering capability, 0.05**°** resolution and ~30 ms steering latency. The angle magnifier module is composed of AC254-100-A-ML (Thorlabs), ACL50832U-A (Thorlabs), and 69856 (Edmund Optics). The communication signal is generated from an arbitrary waveform generator (AWG, M8190A, 5 GHz BW) followed by an amplifier (ZFL2500VH, 2.5 GHz BW). For the OFDM parameters in Eq. (1), there is a fixed parameter (f_clk_ = 8 GHz) which comes from the equipment’s constraint. The other parameters are optimised to achieve the maximum data-rate with BER less than 3.8 × 10^−3^. For instance, for the violet channel in Fig. 3(d), the optimum parameters are found to be N_fft_ = 1024, N_*c*_ = 10, f_os_ = 2 and b_sum_ = 2154, which leads to a data-rate of 8.33 Gb/s.

Band-pass filtering (with Thorlabs DMLP425L, DMLP490L, and DMLP550L) separates the wavelength into four pass-bands (λ < 410 nm, 440 nm < λ < 475 nm, 505 nm < λ < 533 nm and 565 nm < λ). Then, each channel is focused using a high numerical aperture lens, ACL50832U-A (Thorlabs). The signal is detected by a free-space photo receiver with an active area of Φ = 0.4 mm (HSA-X-S-1G4-SI, Femto) followed by an oscilloscope (MSO9254A, 2.5 GHz BW, Keysight), for communication performance tests. The experiment was taken in a low background light condition for a convenience, but it is expected the system can be robust against a normal room lighting due to the WDM pencil beam system structure spatially and spectrally filtering the ambient light.

## Data Availability

Data associated with the figures can be accessed at ora.ox.ac.uk.
